# Therapeutic Effects of Inhaled Nitric Oxide Therapy in COVID-19 Patients

**DOI:** 10.3390/biomedicines10020369

**Published:** 2022-02-03

**Authors:** Nikolay O. Kamenshchikov, Lorenzo Berra, Ryan W. Carroll

**Affiliations:** 1 Cardiology Research Institute, Tomsk National Research Medical Center, Russian Academy of Sciences, 634012 Tomsk, Russia; 2 Department of Anaesthesia, Critical Care and Pain Medicine, Massachusetts General Hospital, Boston, MA 02114, USA; lberra@mgh.harvard.edu; 3 Department of Anaesthesia, Harvard Medical School, Boston, MA 02115, USA; rcarroll4@mgh.harvard.edu; 4 Division of Pediatric Critical Care Medicine, Massachusetts General Hospital, Boston, MA 02114, USA

**Keywords:** nitric oxide, COVID-19, acute respiratory syndrome coronavirus 2, inhaled nitric oxide therapy, endothelium

## Abstract

The global COVID-19 pandemic has become the largest public health challenge of recent years. The incidence of COVID-19-related acute hypoxemic respiratory failure (AHRF) occurs in up to 15% of hospitalized patients. Antiviral drugs currently available to clinicians have little to no effect on mortality, length of in-hospital stay, the need for mechanical ventilation, or long-term effects. Inhaled nitric oxide (iNO) administration is a promising new non-standard approach to directly treat viral burden while enhancing oxygenation. Along with its putative antiviral affect in COVID-19 patients, iNO can reduce inflammatory cell-mediated lung injury by inhibiting neutrophil activation, lowering pulmonary vascular resistance and decreasing edema in the alveolar spaces, collectively enhancing ventilation/perfusion matching. This narrative review article presents recent literature on the iNO therapy use for COVID-19 patients. The authors suggest that early administration of the iNO therapy may be a safe and promising approach for the treatment of COVID-19 patients. The authors also discuss unconventional approaches to treatment, continuous versus intermittent high-dose iNO therapy, timing of initiation of therapy (early versus late), and novel delivery systems. Future laboratory and clinical research is required to define the role of iNO as an adjunct therapy against bacterial, viral, and fungal infections.

## 1. Introduction

The global Coronavirus Disease 2019 (COVID-19) pandemic has become the deadliest respiratory infection since the Spanish Flu in 1918–1920. COVID-19 is a respiratory infection that spans from mild involvement of the upper respiratory tract to severe pneumonia leading to respiratory failure, shock, and death [1].

COVID-19-associated complications are multifactorial and involve endothelial cell dysfunction, local hyperinflammation, intravascular coagulation, and microthrombosis [2,3,4]. This constellation of systemic and organ endotheliopathy has been proposed to explain phenotypic variants associated with COVID-19 [5,6,7,8,9]. A decrease in the angiotensin-converting enzyme 2 (ACE2) activity, caused by its interaction with the SARS-CoV-2 virus and endocytosis of the viral complex and downregulation, leads to an increase in vasoconstriction, pro-coagulation, pro-inflammation, and pro-oxidant angiotensin II effects. These effects, in turn, potentiate inflammation, trigger the release of injury-associated molecules, and cause viral damage to the lung. Specific viral and cytokine storm-induced endothelial damage is called SARS-CoV-2-associated endotheliitis, reflecting COVID-19 microangiopathy in the lungs, and other organs, coupled with the development of thrombosis due to vasculitis.

A respiratory treatment that halts the progression of disease could substantially improve both saved lives and spared hospital resources. At this time, treatment with dexamethasone (a systemic steroid) has been shown to reduce the 28-day mortality in patients requiring mechanical ventilation or oxygen but has no effect on the early progression of the disease [10]. Thus far, no inhaled treatment has been shown to be beneficial to both respiratory and systemic pathophysiology, the endothelium, and viral load, with the potential to halt progression of disease [11].

Antiviral drugs, such as molnupiravir and remdesivir, demonstrated an improvement on mortality, length of in-hospital stay, and the need for mechanical ventilation in specific subpopulations of hospitalized patients only [12]. Despite these advancements in therapeutics, an intervention that readily combines the improvement of clinical symptoms, including hypoxia, and the reduction of endothelial dysfunction is still lacking. High-dose inhaled nitric oxide (iNO) holds promise as an intervention that fulfills these goals.

We present here a review of published studies and ongoing trials on the therapeutic effects of iNO for COVID-19.

## 2. Historical Benefits of NO: iNO as Selective Pulmonary Vasodilator Improving Ventilation/Perfusion Matching

iNO gas is a therapy currently approved for the treatment of pulmonary hypertension in newborns and is also used as a rescue therapy in patients with ARDS [13,14]. Type 3 pulmonary hypertension (due to pulmonary pathology and/or hypoxia) occurs in 40–75% of patients admitted to the ICU with pneumonia, and up to 50% of patients with ARDS [15]. In 21% of ARDS cases, patients develop acute RV failure and acute pulmonary heart disease, associated with an increase in pulmonary vascular resistance [16,17]. During the current COVID-19 pandemic, elevated pulmonary artery systolic pressure has been identified as a hemodynamic parameter associated with a fatal outcome [18]. These data support the use of preventive interventions for RV failure as therapeutic targets and a reframing of therapy for ARDS, adding ‘RV protective’ strategies to the well-established ‘lung protective’ ventilation strategy [17,19].

An ideal medication that optimizes hemodynamics and pulmonary gas exchange should result in improved hypoxemia while positively affecting the underlying pathophysiological mechanisms, thereby preventing fatal complications (reduction of pulmonary hypertension and right ventricular dysfunction). In such a case, targeted antiviral therapy could be coupled with adjuvant pathogenetic therapy directed against the mechanisms that directly lead to mortality. iNO holds promise as an all-encompassing therapy for COVID-19 patients. It is a selective pulmonary vasodilator with known beneficial physiological effects for use in patients with ARDS, potentially reducing the acute RV failure associated with high mortality due to COVID-19 pneumonia, influences the mechanisms that enhance V/Q matching and reduce shunt alteration, and has direct anti-viral effects based on in vitro evidence [20]. The available data on the effect of iNO as a possible adjuvant agent for respiratory treatment in patients with COVID-19 are inconclusive. However, high-quality evidence from past pandemics suggests that iNO use in ARDS, due to SARS-CoV, results in improved arterial oxygenation, decreased pulmonary hypertension, and reduced spread and density of lung infiltrates [21]. However, iNO use has failed to reduce mechanical ventilation days and overall mortality in ARDS patients [21,22,23,24]. Considering the phenotype of intrapulmonary vascular architecture abnormalities found in COVID-19, there is a deep adaptive pathophysiological rationale for the favorable iNO-mediated changes in pulmonary hemodynamics. In COVID-19, an increase in the V/Q ratio is observed in well-aerated, intact sections due to angiotensin II-mediated vasoconstriction, endothelial dysfunction, vasculitis, and thrombosis [25]. Therefore, lung lesions in COVID-19 are not homogenous and occur due to the alternation of the alveolar shunt and alveolar dead space compartments [26,27]. The SARS-CoV-2 virus uses the angiotensin-converting enzyme 2 (ACE2) receptor to enter the cell, which leads to a decrease in its activity, through a negative feedback mechanism, while the levels of plasma angiotensin II increase and angiotensin (1–7) decrease [28]. In this case, a specific pulmonary vasoconstrictor, pro-inflammatory, procoagulant phenotype is formed, mediated by angiotensin II [29,30]. This was confirmed by Santamarina MG et al., who showed that the pathophysiology of moderate to severe respiratory failure in COVID-19 is based on a severe ventilation/perfusion (V/Q) mismatch [8,9]. The authors demonstrated a decrease in the V/Q ratio in areas of the lung parenchyma with ground-glass nodules or consolidation due to an increase in perfusion in poorly ventilated areas. These areas are the morphological basis of the alveolar shunt in conditions of secondary loss of compensatory hypoxic pulmonary vasoconstriction [31].

**Figure 1:**
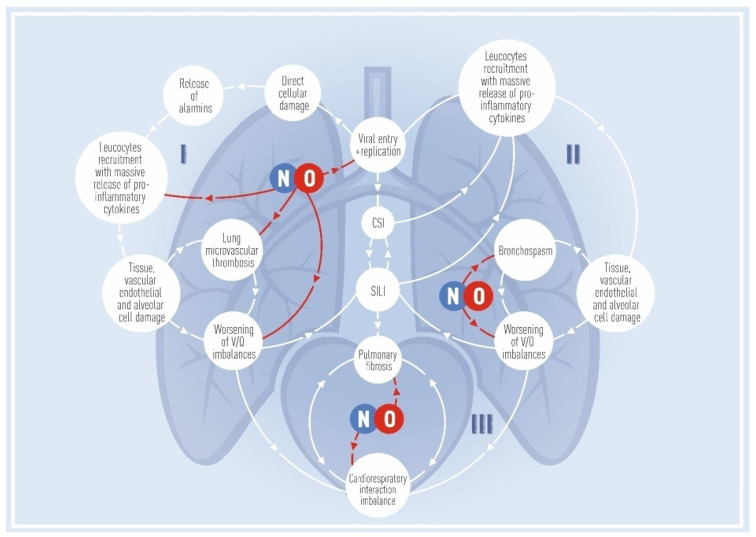
Illustrates the continuum of intrapulmonary pathophysiology at the early stages of COVID-19 pneumonia, highlighting the putative beneficial effects of NO. Part I. The effects of iNO are etiopathogenetic. iNO may reduce viral load while halting the intrapulmonary cascade of inflammation, decreasing alveolar dead space, and thus optimizing ventilation-perfusion. In addition, a decrease in respiratory rate, improvement of gas exchange, and enhanced respiratory comfort may prevent the development of self-induced lung injury (SILI). iNO may also prevent co- and superinfection (CSI), including those involving hospital antibiotic-resistant flora. To this end, it is important to consider administering iNO therapy at the early stage of disease, before the development of irreversible changes in the lungs. Part II. Persistent SILI and CSI contribute to transition of disease into a self-maintaining and self-sustained process. The continuum of intrapulmonary pathophysiology is mediated by local and systemic hyper-inflammatory reactions, even after virus elimination. There is an increase in elastance and a decrease in aerated lung, an increase in intra-alveolar exudation, a transition to low compliance phenotype, mirroring the known pathogenesis ARDS. The NO-mediated impact at this stage is aimed at optimizing V/Q matching. Considering the transition of functional changes in the lungs to morphological changes, therapeutic effects of iNO would be quite limited, represent last-resort treatment, and do not consistently result in improved outcomes. Part III. iNO-mediated cardio-respiratory interactions to reduce the risk of right ventricular failure. Prevention of acute cor pulmonale development by reducing right ventricular afterload. Prevention of group 3 pulmonary hypertension due to prolonged antifibrotic effects of iNO in the lungs.

At the same time, an increase in the V/Q ratio is observed in well-aerated intact sections due to angiotensin II-mediated vasoconstriction, endothelial dysfunction, vasculitis, and thrombosis [25]. Therefore, lung lesions in COVID-19 are inhomogeneous and occur due to the alternation of the alveolar shunt and alveolar dead space compartments [27]. Mechanical ventilation and other respiratory support strategies (non-invasive ventilation and high-flow oxygen therapy) do not affect the V/Q ratio in intact ventilation and perfusion pathology, while the positive effects of prone position on oxygenation can be explained by the gravitational redistribution of blood flow to intact areas with high V/Q ratio and a cumulative V/Q optimization in general. At the same time the increase in alveolar dead space is independently associated with mortality in ARDS [32]. This pathophysiological theory supports the early use of pulmonary vasodilators, such as iNO (Figure 1) [8,9]. To this end, there is evidence that iNO may be the most effective in patients with COVID-19 respiratory failure and concomitant pulmonary hypertension [33,34].

## 3. Burgeoning Antimicrobial Effects of iNO-Therapy: Viral, Bacterial, Fungal

The clinical role of NO in COVID-19 patients may be relevant due to in-vitro evidence of NO antiviral activity, specifically against the SARS coronavirus [35,36]. Preclinical and clinical evidence suggests that iNO has a viricidal effect, including the family of Coronaviridae. Moreover, in vitro studies demonstrate that the use of NO-donor compound S-nitroso-N-acetylpenicillamine leads to an increased survival rate of in vitro mammalian cells infected with SARS-CoV. SARS-CoV and SARS-CoV-2 share the same subgenus within the family Coronaviridae. Recent studies have confirmed that NO can inhibit SARS-CoV-2 replication, and the main viral protease for targeted NO therapy has also been identified [37].

Similar therapeutic effects of NO can be expected in patients with COVID-19 due to the genetic similarities between the two viruses. The literature seems to point towards nonspecific rather than pathogen-specific antimicrobial effects of NO. Thus, the role of exogenous iNO as an antiviral agent during a COVID-19 infection could be hypothesized [38].

NO-dependent virus elimination is most likely mediated by inhibiting viral replication with genetic modification [39,40,41,42,43,44,45,46,47,48,49,50,51,52,53]. Antiviral defense mechanisms involve the deactivation of viral proteins essential for viral replication: viral proteases, reverse transcriptases, transcription factors, etc. mediated by S-nitrosylation of essential thiol groups [54]. Dinitrosyl iron complexes with thiol ligands in lung and airway tissues, created by high-dose iNO therapy due to the prolonged release of nitrosonium cation (NO+) donors can suppress various metabolic processes utilized by a coronavirus that are responsible for penetration into cells as well as replication [55,56]. A. F. Vanin et al., using an experimental model and healthy volunteers, demonstrated that the inhalation of high-dose NO could be followed by the absorption of a large portion of the agent in the lung and respiratory tissues as dinitrosyl iron complexes with thiol ligands [57]. Systemic side effects did not occur. The authors concluded that a high concentration of NO donors are the main sites that host coronavirus in the human body as a result of the contact with a high-concentration of iNO and, therefore, could be of great use during eradication of a coronavirus [57]. High-concentrations of NO have been found to be microbicidal but still safe in spontaneously breathing subjects in a phase I trial [58]. However, the optimal therapeutic regimens and the efficacy of NO gas in reducing viral load while improving oxygenation in hypoxemic COVID-19 patients have not yet been tested. A pilot study showed that low-dose iNO (max 30 ppm) could shorten the time of ventilatory support for patients infected with SARS-CoV [21].

The antimicrobial and antifungal effects of iNO, convincingly shown in a number of studies, can improve the clinical course of COVID-19 in conditions of co- and superinfection [59,60]. The detection rate of atypical respiratory pathogens and viral co-infections in the general population of hospitalized patients with COVID-19 has reached 15.6–20.7% [61,62]. Despite the low incidence of bacterial co-infection upon admission to the hospital, 14–100% of patients admitted to ICUs demonstrate a co-infection, including bacterial co- and superinfection [63,64], while the frequency of ventilator-associated pneumonia (VAP) in a SARS-CoV-2 infection has been reported to be 50.5% [65]. VAP is associated with an increased 28-day mortality rate and longer duration of mechanical ventilation and ICU length of stay in SARS-CoV-2 patients [66]. Fungal invasion is also a major problem in the management of COVID-19 patients, which, together with bacterial infections, can significantly increase therapy costs and possibly worsen outcomes [67]. Thus, the incidence of secondary infections in patients hospitalized with COVID-19, especially in the ICU, may not be as low as that in early reports, and the iNO therapy may be able to improve outcomes by expanding the anti-infective spectrum of therapy regardless of the etiologic agent.

The type of escalation therapy, aimed at a wide range of pathogens, is especially promising in conditions of scarcity of resources for accurate verification of an infectious agent and determination of its possible resistance to therapy, including antibiotic resistance. In addition, the current principles of therapy for a complicated disease course of a SARS-CoV-2 infection prescribe immunosuppressive drugs as therapy for the “cytokine storm”, which can lead not only to secondary infections, but also contribute to the long-term persistence of the virus in the body, and increase the frequency of its mutations and the likelihood of emerging new strains. In this regard, the early use of iNO as a universal direct-acting antiviral agent also seems reasonable. It could reduce viral load and attenuate the direct damaging effect of the virus on the lungs. The adjuvant antibacterial and antifungal effects of iNO could prevent the development of secondary infections of the respiratory tract, which is especially important in conditions of the multidrug-resistant bacteria commonly found in the ICU.

At the time being, iNO adjuvant therapy is recommended by a number of researchers as an effective strategy for the treatment of COVID-19 [68,69,70].

## 4. Other Benefits of iNO-Therapy: Endothelial Stability, Improving Inflammation, Biofilm Dispersion

Systemic and organ endotheliopathy in COVID-19 is likely multifactorial and involves direct viral invasion, endothelial cell dysfunction, local hyperinflammation, intravascular coagulation, and microthrombosis [2,3,4]. iNO therapy may reduce endothelial pulmonary syndrome with microvascular thrombosis. Possible favorable systemic actions of iNO treatment include antiplatelet, leukocyte antiadhesive, and anti-inflammatory effects, which can potentially prevent a cytokine storm and reduce the risk of extrapulmonary organ complications [71]. Moreover, the presence of a reserve pool of NO in extrapulmonary tissues due to the S-nitrosylation mechanism leads to a decrease in vascular tone and reduces the risk of thrombosis and leukocyte adhesion to the systemic vascular endothelium [72]. Severe forms of COVID-19 are associated with exaggerated and persistent injury to the endothelium of pulmonary microvasculature [73], endothelial apoptotic bodies were also observed during an autopsy in other organs [74]. In addition to bronchial epithelial cells and pulmonary type II cells, the SARS-CoV-2 virus has a tropism for endothelial cells leading to cell apoptosis and a decrease of endothelial NO production [75]. A significant decrease in endothelium-dependent synthesis has been demonstrated in patients with COVID-19, especially in complicated cases [76], which led to the hypothesis that NO imbalance is associated with lung damage [77]. Researchers put forth more pathways for the formation of NO deficiency in an infection caused by SARS-CoV-2: NO imbalance, reactive oxygen species associated with dysregulation of angiotensin II-angiotensin (1–7) [78], systemic inflammation, tissue inflammation, mitochondrial dysfunction, and changes to vascular tones by increasing intracellular calcium concentration and reducing the bioavailability of NO [79]. Hypertension, diabetes, and cardiovascular diseases significantly aggravate the course of COVID-19 and are associated with endothelial dysfunction and decreased endothelial NO production and bioavailability [80].

The mechanism for increasing the bioavailability of NO upon its exogenous administration is protein S-nitrosylation and an increase in the concentration of serum NO-NOx metabolites (nitrates, nitrites, S-nitrosothiol, N-nitrosamine, etc.), which serve as reserve NO donors in the body [81,82]. Extrapulmonary accumulation of these metabolites in target organs can realize the distant organ protective effects of exogenous NO [83]. Exposure and stabilization of endothelium function with iNO therapy can reduce COVID-associated distant complications such as AKI [84,85].

In addition, NO has a whole range of important pathogenetic effects: NO can reduce inflammatory cell-mediated lung injury by inhibiting neutrophil activation and local pro-inflammatory cytokine release, leading to a decrease edema in the alveolar spaces [71]. iNO is considered a pharmacological tool for monitoring the excessive inflammatory response of the host organism and overcoming the “cytokine storm” associated with viral diseases [86]. Progressive immunothrombosis in COVID-19 is associated with NO deficiency [87]. Endogenous NO, produced in the paranasal sinuses, and exogenous iNO have bronchodilatory effects and activate mucociliary clearance [88,89,90,91,92]. The enterosalivary nitrate–nitrite–NO pathway, as a storage pool plays an essential role in the prevention of ischaemic cardiovascular and septic events, and deficiency of a synthase-dependent pathway generating NO may be associated with sepsis, organ failure, and an increased mortality rate [92]. Thus, a number of researchers suggest that iNO is essential for sanogenesis of the respiratory tract in various pathological processes, including infections [93,94,95].

Increased salivary and serum NO levels are associated with survival in ARDS caused by the H1N1 virus [96]. There is an alternative point of view suggesting that the NO signaling pathway does not only fail to improve the outcomes of viral infections but can also be an independent damaging factor and/or a marker of the severity of lung damage in some cases [97,98,99,100].

At present, most data highlighting the antimicrobial efficacy of iNO were obtained on the culture of planktonic free forms of bacteria associated with the dispersed phase of the life cycle, which is necessary for the expansive colonization of new habitats. However, in nature, most bacteria live mainly in the heterogeneous multicellular communities of biofilms. The biofilm matrix reduces the degree of penetration of antibiotics, accumulating factors of specific and nonspecific resistance to cells of the immune system and biocides [101,102,103]. In biofilms, there is a modification of the native genetic material and post-transcriptional changes in the phenotype caused by the commensal community [104]. This gives bacterial colonies significant resistance to external factors, forms antibiotic resistance, and allows the avoidance of immune surveillance [105]. Moreover, some antibiotics can cause increased biofilm formation [106].

The proportion of infections associated with biofilm formation in clinical practice reaches 80% [107]. This is associated with resistance and chronicity of pathological process, an increase in morbidity and mortality [108]. Currently, iNO is being viewed as a novel therapeutic strategy for controlling and overcoming biofilm resistance [105]. The use of low doses of NO stimulates the transition to the dispersion phase of the cycle, which leads to the restoration of the sensitivity of biofilms and free bacteria to the action of antimicrobial drugs and increases the efficacy of antibiotic therapy [109], and an increase in exposure time inhibits biofilm formation [110,111,112]. At the same time, the intermittent effect of high concentrations of NO has a direct destructive effect on biofilms of gram-negative and gram-positive bacteria, including nosocomial pathogens [113,114,115]. Clinical studies of iNO use in biofilm-related infections have demonstrated significant reductions in the number of bacterial biofilm aggregates and improvement in lung function, suggesting that the use of iNO as adjunctive therapy may be very beneficial [116]. Widespread adoption of iNO therapy could provide unprecedented control and treatment of biofilms in the current pandemic, improve short-term outcomes for COVID-19 patients, and reduce the probability of “chronic critical illness” and post COVID-19 long-term antibiotic resistance substrate formation [117].

## 5. iNO and COVID-19 Specific Data

An effective etiological treatment for COVID-19 has not yet been developed. The large mortality rates in COVID-19 patients requiring mechanical ventilation are prompting clinicians and scientists to seek new technologies and pharmacological interventions that can improve outcomes. Researchers and clinicians consider iNO therapy promising for patients with COVID-19 and respiratory failure [38,71], supported by in vitro research from Akaberi et al. [37]. In 2003, during the SARS epidemic in China, a small observational study of patients with SARS pneumonia receiving non-invasive support, biphasic positive airway pressure (BiPAP), were treated with NO gas which improved oxygenation, accelerated the resolution of chest X-ray infiltrates, reduced the need for intubation, and led to a more rapid and sustained ARDS resolution and improved overall clinical outcomes [21]. In the current global pandemic, iNO therapy is accepted by the societies of critical care medicine as a temporary measure to maintain or improve oxygenation in mechanically ventilated patients [118,119]. In healthcare practice, about 30% of patients with severe C-ARDS have received iNO as a life-saving therapy [120,121]. However, the results of published randomized trials and clinical observations are highly controversial. Small cohort studies have not demonstrated a significant improvement in oxygenation and clinical outcomes with iNO therapy [122]. On the other hand, the frequency of responders ranges from 25% to 40% with a tendency of a more pronounced effect on gas exchange in patients with right ventricular dysfunction. The percentage of iNO responders is much lower than in patients with non-C-ARDS [123,124].

Several trials tested the efficacy of iNO therapy aimed at improving the outcomes in COVID-19 patients. A retrospective observational study showed that NO gas is useful in improving the oxygenation in spontaneously breathing patients with COVID-19 pneumonia [125]. High-dose nitric oxide (160 ppm) was safely administered to pregnant females with severe COVID-19 pneumonia [126] and as a rescue therapy to spontaneously breathing patients with COVID-19 and hypoxemic respiratory failure [127]. A recent trial non-invasively treating patients with moderate COVID-19 hypoxia demonstrated that iNO-therapy produced an acute improvement of systemic oxygenation in hypoxemic patients and reduced the respiratory rate [128]. Furthermore, data demonstrate that the administration of iNO improves functional status in ambulatory patients [33,34].

Preliminary data support the iNO-mediated improvement of oxygenation in mechanically ventilated patients and spontaneously breathing patients with COVID-19 [129]. However, the optimal therapeutic regimen of iNO administration in spontaneously breathing hypoxemic patients has not been identified.

Another strategy for iNO administration in COVID-19 involves the potential for selective pulmonary vasodilation to optimize ventilation/perfusion matching by reducing pulmonary vascular resistance and decreasing alveolar dead space. The drug intervention scheme herein assumes administration of a long-term, constant NO insufflation at low doses, in contrast to the pulse therapy, aimed directly at the elimination of the viral agent from the respiratory tract. Prolonged iNO therapy can also have an independent moderate antiviral effect, and, due to prolonged exposure, it can reduce inflammatory cell-mediated lung injury by inhibiting neutrophil activation and subsequent pro-inflammatory cytokine release. The study of iNO treatment in spontaneously breathing COVID-19 patients demonstrates not only an increase in oxygenation in all non-intubated patients, but also provides evidence that iNO therapy may have a role in preventing progression of hypoxemic respiratory failure [125].

## 6. Modality of iNO Therapy: Timing of iNO Administration

Hypoxemia in the early stages of COVID-19 is caused by a dysregulated pulmonary perfusion [130,131]. Changes in pulmonary biomechanics demonstrate that at an early, well-defined stage of COVID-19 disease (between the admission to the high-dependency unit to the time of healing or admission to the ICU), the lung weight in C-ARDS was approximately half of what has been described in a typical ARDS. The C-ARDS gas-volume was two times greater than what has been consistently described in the typical ARDS [132]. Thus, in the early stages, C-ARDS is fundamentally atypical, as the severity of hypoxemia is unrelated to the severity of the anatomical lung pathology. Moreover, the authors do not exclude that other viral pneumonias present similar characteristics [132]. In all of the studies that did not show improvement in oxygenation and/or clinical outcomes, attention was drawn to a significant time delay from patient intubation to initiation of iNO therapy, which is largely due to the behavioral despair paradigm of utilizing selective pulmonary vasodilators in severe hypoxemic respiratory failure. The median time from intubation to initiation of iNO, reported by different clinics, ranged from 3.6 days to 6.5 days in studies where iNO was used as a second-line pulmonary vasodilator [122,133]. It is noteworthy that in these cases, the frequency of response to iNO was 64%. Data from other authors indicate a response to iNO therapy in more than 65% of patients with C-ARDS, manifested in a significant increase in the PaO_2_/FiO_2_ ratio reduction in the oxygenation index and a reduction in the dead space fraction, associated with higher baseline BNP and troponin values. The authors put forward a hypothesis about a specific pulmonary vascular phenotype in iNO responders and a special role of pulmonary vascular function in COVID-19 pathophysiology [134,135]. A potential cause of the mentioned dissonance in the obtained data can be the severity and irreversibility of morphological changes both in the lung parenchyma and in the capillary bed of the pulmonary microcirculation at the late stages of the disease: pulmonary oedema and pulmonary consolidation prevent from alveolar supply with iNO; intravascular coagulation and organized thrombosis do not allow the effects of iNO in ventilated alveoli to reduce dead space in severe C-ARDS [25]. Thus, considering the pathogenesis of lung capillary injury and extrapulmonary complications in COVID-19, the use of iNO can be promising at the early stages of the disease when the disorders are potentially reversible or “functional” in nature [136]. De Grado et al. [133] observed a trend towards higher tidal volume and compliance in individuals who responded to inhaled vasodilators therapy, which led the authors to suggest that inhaled vasodilators therapy would be more effective in patients with a sufficient volume of functioning alveoli, possibly at the early stages of the disease before the evolution to late stage ARDS with low-compliance [27], while other authors suggest concentrating on the “vasocentric” nature of this disease [137]. We support the idea of tailoring the initiation of iNO therapy, which must be correlated with the stages of the progression of the disease-related intrapulmonary aberrations. Early initiation of iNO therapy may alter the dramatic development of COVID-19 pneumonia pathophysiology.

Figure 1 illustrates the evolution of COVID-19 from early to late stages and demonstrates the points of application of iNO (Part I: early stage; Part II: late stage; Part III: interaction in the lung-heart system).

The use of iNO in the early stages of the disease has pluripotent effects and can help eliminate the pathogen, stabilize and reduce pulmonary stress associated with changes in transpulmonary pressure in dyspnea, improve gas exchange, and prevent a secondary infection. Ultimately, this may prevent disease progression, and iNO should be considered as an intervention with self-healing potential. The use of iNO in the late stages of COVID-19 at severe extensive morphological changes in the lung parenchyma (usually consolidation) is an exclusively temporary measure to maintain gas exchange before escalation therapy (for example, transition to ECMO). In terms of cardio-respiratory interactions, the use of iNO may reflect the new concept of ‘RV protective’ strategies for respiratory therapy in the short and long term (prevention of acute cor pulmonale and chronic group 3 pulmonary hypertension).

## 7. Modality of iNO Therapy: Dosage Mode (Continuous versus Intermittent iNO Administration)

The idea of intermittent dosing of high-dose iNO to combat a SARS-CoV-2 infection appeared after analyzing data on COVID-19 cases among smokers in different countries: the incidence of COVID-19 patients among smokers was lower than in the general population [138,139,140]. While the toxic effects of smoking are devastating, it is worthy to note here for the purpose of this review that NO ranges in each puff from 250–1350 ppm, which is much higher than the medical use of iNO, generally no more than 80 ppm [141,142], suggesting that iNO dosed in short bursts and at high concentrations may protect against COVID-19 [143].

High doses of NO have been shown to be safe and well tolerated (160 ppm for 30 min, 5 times a day for 5 days) in healthy volunteers [58]. The original system for the high-dose iNO delivery for hospitalized patients was developed by Gianni S et al. [144]. It was easy to use and safe in clinical practice. A small group of pregnant patients (*n* = 6) with severe and critical COVID-19 received NO inhalation by mask twice a day at a high dose (160–200 ppm) for 30 min [126]. The authors explain the reasoning for this compassionate-use intermittent therapy, including its potential antiviral, anti-inflammatory, and mild bronchodilatory effects, in addition to selective pulmonary vasodilation, which may improve maternal and fetal oxygenation. Furthermore, iNO therapy was shown to be well-tolerated and associated with improved oxygenation, respiratory rate, cardiopulmonary system function in this population [126]. Moreover, in some patients, there was an association between intermittent high-dose iNO therapy and a decrease in markers of systemic inflammation [126]. Early adoption of this system contributes to a decrease in the respiratory rate, enhances patient respiratory comfort, and reduces the work of breathing. It might also prevent transpulmonary pressure variations and the progression of self-induced lung injury. iNO-mediated bronchodilation and the improvement of bronchial patency also prevent atelectasis formation, minimizing a decrease in lung compliance and progression to lung fibrosis. This hypothesis is supported by the encouraging results of clinical observations and studies carried out in spontaneously breathing patients with COVID-19-associated hypoxemic respiratory failure. Fakhr B.S. et al. demonstrated that the administration of iNO at 160 ppm for 30 min twice daily promptly improved the respiratory rate of tachypneic patients and systemic oxygenation of hypoxemic patients. No adverse events were observed [128]. None of the subjects were readmitted or had long-term COVID-19 sequelae [128]. Similar results were obtained in a group of COVID-19 patients at high risk for acute hypoxemic respiratory failure with worsening symptoms, despite the use of supplemental oxygen and/or awake proning, who were treated with high-dose iNO [127]. NO pulse therapy has been stated to be well-tolerated and used as an effective adjuvant rescue therapy for patients with COVID-19 [127]. Moreover, the authors suggest its effectiveness as an alternative to available antiviral drugs in cases when they may be contraindicated (pregnancy, drug intolerance, etc.).

Parikh et al. used the strategy for continuous iNO therapy in spontaneously breathing patients with COVID-19; at the same time, 54% did not require invasive mechanical ventilation after treatment with iNO [125]. If these findings are confirmed in larger studies, treatment with iNO, along with high-flow oxygen therapy and non-invasive ventilation may become the first respiratory treatment for this large cohort of patients.

A clinical trial titled “Nitric oxide therapy for COVID-19 patients with oxygen requirement (NICOR)” was registered with ClinicalTrials.gov (#NCT04476992). The trial aims to study the safety of intermittent versus continuous iNO in spontaneously breathing COVID-19 patients. Authors hypothesize that high-dose iNO with an adjunct of continuous low dose administration between the high-dose treatments can be safely administered in hypoxemic COVID-19 patients compared to the high dose treatment alone. The potential benefits of the prolonged administration may reduce the severity of disease and time to recovery in COVID-19 patients. Together, with a prolonged clinical effect on ventilation-perfusion matching, a prolonged regimen might increase antiviral activity (dose and time-dependent). In this trial, the iNO spike therapy aims at suppressing replication and eliminating the virus. The constant iNO delivery aims to reduce pulmonary vasoconstriction and optimize V/Q matching. There is an observation that the mass of well-aerated lung tissue decreases over time, which makes it impossible to deliver iNO to the alveoli and thus underscores the importance of early therapy. Lung inhomogeneity manifests in mosaicism of areas of hyperperfusion, hypoperfusion, and consolidation, as a morphological substrate for a combination of continuous and intermittent iNO therapy. At the same time, it is necessary to emphasize the critical importance of early initiation of therapy during potentially reversible phases of lung injury, ideally before the consolidation occurs, which radically changes the current paradigm of the iNO application. Though essential, the study #NCT04476992 has some limitations as it is a single-center study on a limited population of patients of the same ethnicity. Patients enrolled in the study needed oxygen therapy but were without severe gas exchange abnormalities as they were treated in the hospital general medicine ward, and the proportion of older adults, comorbidity and frailty in the study were low. Patients with severe C-ARDS might have a different respiratory pattern and physiological response to iNO treatments. Nevertheless, this study has been the largest randomized analysis of different iNO treatment options in spontaneously breathing COVID-19 patients; it has now been completed and its results have been prepared for publication, but larger scale studies are required.

## 8. Special Consideration of Safety iNO in COVID-19

There are a number of areas of potential concern with iNO therapy, especially with high-dose regimens: (1) direct toxicity; (2) toxicity associated with the oxidative product; NO_2_; (3) the formation of high concentrations of methemoglobin; (4) the possibility of rebound pulmonary hypertension; (5) systemic hemodynamic disorders; (6) decreased platelet activation and subsequent aggregation; and (7) increased risk of acute kidney injury [145].

Direct iNO toxicity may, in fact, contribute to bacterial death when high-dose iNO is used as an antimicrobial. Traditionally, the concern for direct iNO toxicity stems from high doses inhaled by the smoker population. As researchers investigate the benefits of high-dose iNO, one of the first steps will be to determine the least toxic, effective dose. Intertwined with determining the safety of high-dose iNO, there is a known potential of inhaling the byproduct of the interaction of NO with oxygen: nitrogen dioxide, NO_2_. NO is highly reactogenic; in the presence of oxygen (O_2_), it undergoes a chemical reaction with the formation of NO_2_: 2NO + O_2_ = 2NO_2_. NO_2_ combines with water to become nitric acid, which can be caustic to tissues. NO_2_ itself is a highly non-toxic gas with a maximum permissible level of 5 ppm as a short-term exposure and 3 ppm in an 8-h time-weighted average, and more conservative recommendations regulate a short-term exposure limit of 1 ppm [146]. The level of NO_2_ in the gas mixture delivered to the patient must be monitored continuously throughout the entire period of NO therapy. The rate of NO_2_ formation depends on the concentration of NO and O_2_, the time during which the two gases come in contact, pressure, and temperature. This fact has important implications for NO delivery: sources with high concentrations of NO should be avoided, and NO and inspiratory O_2_ should be used in minimal, clinically acceptable doses. Monitoring NO_2_ during iNO therapy is absolutely essential, and the addition of “scrubbers” to the ventilator circuit can be used to absorb NO_2_, including charcoal and lime soda [142,144]. Air, or N_2,_ may be used as a diluent and is required when high levels of NO are administered [147]. Available in stock iNO delivery systems are equipped with alarms when the level of NO_2_ has reached upper limits.

NO oxidizes hemoglobin from the ferrous (Fe^2+^) to the ferric (Fe^3+^) form, rendering the hemoglobin incapable of attaching and delivering oxygen. Therefore, when delivering high-dose iNO, the level of methemoglobin (MetHb) in blood/plasma, invasively or non-invasively, should be monitored. The level of MetHb present during inhaled NO therapy depends on the amount of MetHb formed from oxidation and the amount eliminated by reduction within erythrocytes by the methemoglobin reductase enzyme. In most clinical situations, MetHb levels remain low during the NO therapy. The maximum level in clinical practice should be kept to less than 5% of the total hemoglobin concentration, while it can be monitored both discretely with blood sampling and/or continuously and non-invasively using pulse co-oximetry technology. MetHb monitoring is especially important in patients with hypoxemia and/or a suspected methemoglobin reductase enzyme defect. Prior studies using intermittent high-dose iNO have demonstrated safe metHb levels [126,127,128,148].

Abrupt discontinuation of iNO may be accompanied by rebound pulmonary hypertension, characterized by worsening oxygenation and hypoxemia, systemic hypotension, bradycardia, and acute right ventricular failure [149]. However, these phenomena were noted only after the discontinuation of long-term continuous therapy with iNO, and there have been no reports of this in the current pandemic; timely diagnosis of this condition comes down to careful monitoring of the patient. Earlier studies using high-dose NO have not demonstrated rebound pulmonary hypertension [148].

The danger of a decrease in platelet activation and subsequent aggregation during iNO therapy was not demonstrated in a randomized, controlled, blinded study on the hemostasis in healthy adults after being administered iNO [150], and in the case of COVID-19, earlier studies have not revealed a worsening of hemostasis [127,128,129].

A significant limitation to the widespread administration of iNO therapy could be the fear of an increased risk of acute kidney injury seen in patients with non-COVID ARDS [151], but our data does not confirm this, and in fact, refutes this notion [152]. Taking into consideration the uniqueness of the pathogenesis of organ complications in COVID-19, the data obtained may indicate the possibility of context-sensitive use of iNO in various clinical scenarios, in particular, to reduce microvascular damage and microcirculatory thrombosis, leading to systemic manifestations of infection and organ dysfunction [75]. iNO therapy may be the key to preventing angiocentric multi-organ injury, unique to COVID-19, caused by endothelial cell injury and the development of systemic “vascular disease” [153]. In systemic endothelial dysfunction, the deficiency of the synthase-dependent pathway of NO generation can be corrected by its exogenous supplementation. Recent studies reported a decreased total NO and its metabolites in hospitalized COVID-19 patients, as well as a decreased nitric oxide diffusion in around 40% of patients discharged from the hospital [154,155]. Other authors claim that reduced concentrations of NO metabolites may be potential biomarkers of long-term poor or irreversible outcomes after a SARS-CoV-2 infection and might serve as a predictor to track the health status of recovered COVID-19 patients [156].

However, to confirm or refute this hypothesis, further studies of NO homeostasis in COVID-19 are needed within the framework of the classical and non-enzymatic pathways of its synthesis and exchange. Endogenous NO metabolism and the role of endothelial and inducible NO synthases in COVID-19 require further research. It is especially important in the context of safety, since one of the mechanisms for the development of acute kidney injury during iNO therapy may be oxidative stress associated with overexpression of iNOS and the overproduction of endogenous NO in the case of non-COVID ARDS [157].

The unique nature of systemic inflammation and the cytokine profile in COVID-19 suggests a different pathway from classic non-COVID ARDS mechanisms of extra-pulmonary organ dysfunction. In particular, the level of cytokines, even in severe and critical COVID-19, is significantly lower than in other disorders associated with lung injury (hypo- and hyperinflammatory ARDS, sepsis, etc.) [158]. It will be important to better understand the imbalance of endogenous NO production in the context of C-ARDS.

In conclusion from a safety profile, a low and high dose of iNO can be safely implemented if continuous monitoring of metHb, NO_2_, and O_2_ levels, as well as the use of NO_2_-scavengers, are diligently employed.

## 9. Challenges and Innovations: iNO Delivery Devices

The cost of iNO treatment is an essential factor to consider, especially when iNO is administered over days or in the context of high-dose therapy. The reported cost of the NO is $6/L [159], at the same time, the average cost of 1 h of therapy is approximately 100–150$ [160,161]. Annual clinic costs associated with iNO were reported approximately as high as $1.8-million, and nationwide, this could be approximately $200 million, with analysis only available for the neonatal patients [162]. Naturally, financial costs and logistical challenges make it impossible to widely implement iNO therapy to combat the current COVID-19 pandemic. In this regard, it is extremely important to introduce it into clinical practice using novel delivery devices. Currently, there is an active development of new iNO delivery devices that offer portability, stability, and on-demand NO generation. The essence of the development is to abandon traditional cylinder-based systems and switch to bedside NO synthesis and delivery technologies: electricity-generated NO systems, chemical-based NO systems, NO-releasing solutions, and nanoparticle NO technology. These devices are at different stages of development: from clinical testing to the start of sales in some countries. A detailed overview of the systems currently developed to administer inhaled NO for mechanically ventilated and non-intubated patients is presented by Gianni S, et al. [163]. It should be noted that a study on the comparison between high-dose nitric oxide delivered from pressurized cylinders and nitric oxide produced by an electric generator from air has already been carried out and demonstrated a high efficacy and safety of the technology [164]. Completion of the current clinical trials, FDA and European regulatory approval, and market entry into the commercial sector of these devices can revolutionize iNO delivery in medicine and, in particular, with COVID-19. For these purposes, an inhalation mask system to deliver high concentrations of iNO in spontaneously breathing subjects has already been developed [144,165].

## 10. Future Directions

Every year, new strains of viruses with pandemic potential appear in the world. Antibiotic resistance of known microorganisms increases. The planet’s population is rapidly aging, and the population’s polymorbidity is increasing. New pandemics are likely to await humanity, and in order to overcome them, healthcare systems need to develop a coordinated response strategy to face the significant uncertainty in the etiotropic therapy of pathogens and pathogenetic approaches. iNO can significantly change the trajectory of this response both in the current pandemic and in future scenarios. Clinicians’ and researchers’ efforts should be combined to define the antimicrobial activity of iNO. Possible prospects for further research are presented in Table 1.

## 11. Conclusions

The review of iNO impact on COVID-19 pathophysiology and approaches to iNO delivery suggest that the early administration of iNO therapy may be a safe and promising approach for treatment of COVID-19 patients and beyond. Future large studies focusing on safety and efficacy of iNO therapy regimens in patients with hypoxemic respiratory failure associated with COVID-19 or other viral infections, are required and should be based on the traditional safety paradigm of iNO therapy. For the widespread introduction of iNO therapy into clinical practice, fundamental studies of homeostasis, metabolism and bioavailability of endogenous NO in COVID-19 are needed. The evaluation of endothelial and inducible NO synthases is extremely important for the development of personalized therapeutic protocols, as well as risk stratification and prognosis of severe disease in COVID-19 positive patients.

## Figures and Tables

**Table 1:** Prospects for further research investigating the beneficial effects of iNO.

**Local Effects in the Lungs**
Optimization of V/Q matching: electrical impedance tomography, CT angiography
Anti-inflammatory and antiproliferative effects: concentration of inflammatory mediators in bronchoalveolar lavage, pulmonary ultrasonography, CT scan
Antiviral effects: *viral load*, PCR cycle time
Effect of NO-therapy on the microbiome of the respiratory tract, frequency of superinfections and secondary infectious complications
Effect of NO-therapy to prevent disease progression: reduction in intubation frequency, reduction in duration and aggressiveness of respiratory therapy
Impact on long-term pulmonary function (“long COVID”): level of reducing fibrotic lung disease after C-ARDS
**Systemic Effects (Nitrosylhem Formation)**
Anti-inflammatory effect: concentration of interleukins and inflammatory markers in the peripheral blood, improvement of organ function
Antiplatelet effect: D-dimer, thromboelastography, thromboembolic burden, improvement of distal organ function (e.g., AKI, liver function)
Suppression of apoptosis: long-term improved organ functions, improved long-term clinical outcomes
Influence on the general functional state and the degree of frailty of patients in the long-term period after suffering from COVID-19: KATZ score
**Individual and Population Effects**
Expression of inducible and endothelial NO synthases and metabolism of endogenous NO in COVID-19 patients
NO-therapy in patients of various COVID-19 endotypes: thrombotic, immunopathic, adaptive
NO-therapy in specific categories of patients with COVID-19 and comorbidity, increasing the risk of a severe course of the disease: chronic lung disease; conditions associated with endothelial dysfunction: hypertension, diabetes mellitus, obesity, smoking
Optimal start time of NO-therapy and its variant (intermittent versus intermittent + continuous inhalation): optimization to the phase of the disease course and individual trajectory (possibly not only by clinical markers of hypoxemia development, but also by laboratory indicators of disease progression, for example, D-dimer)
The effect of adjuvant NO-therapy on mutagenic activity of the virus: sequestration of the virus genome in individuals and in the population
NO therapy and the development of antibiotic resistance in individuals and the population

## Data Availability

The datasets used and/or analyzed during the current study are available from the corresponding author on reasonable request.

## Abbreviations

AHRF	Acute Hypoxemic Respiratory Failure
iNO	Inhaled Nitric Oxide
ICU	Intensive Care Unit
ARDS	Acute Respiratory Distress Syndrome
RV	Right Ventricular; V/Q: Ventilation/Perfusion
ACE_2_	Angiotensin-Converting Enzyme 2
AKI	Acute Kidney Injury
C-ARDS	COVID-Related Ards
PaO_2_/FiO_2_	Arterial Oxygen Partial Pressure/Fractional Inspired Oxygen
MetHb	Methemoglobin
